# A Change

**Published:** 1884-02

**Authors:** 


### A CHANGE.

Dr. Thomas Fillebrown, of Portland, Maine, has removed to Boston, and may henceforth be found at Hotel Bristol, Boylston and Clarendon Streets. He has been elected to the chair in Harvard lately occupied by Dr. L. D. Shepard, whose professional engagements no longer permit him to fulfill the duties of the position. Prof. Fillebrown is a worthy successor to one who has so long and ably filled the chair of Operative Dentistry at Harvard.

